# Pneumopericardium and multicompartmental air dissemination after colorectal endoscopic resection managed conservatively: a case report

**DOI:** 10.3389/fmed.2026.1848086

**Published:** 2026-07-22

**Authors:** Zhiyang Wu, Juan Zhang, Xingsheng Xu, Zhi Li

**Affiliations:** 1 Department of Critical Care Medicine, Qilu Hospital (Qingdao), Cheeloo College of Medicine, Shandong University, Qingdao, Shandong, China; 2 Department of Ophthalmology, Qingdao Women’s and Children’s Hospital, Qingdao, Shandong, China; 3 Department of Cardiology, Qilu Hospital (Qingdao), Cheeloo College of Medicine, Shandong University, Qingdao, Shandong, China

**Keywords:** case report, CO_2_ insufflation, endoscopic resection, iatrogenic perforation, nitrogen washout, pneumopericardium

## Abstract

**Background:**

Colonoscopy-associated perforation is a recognized complication of therapeutic lower gastrointestinal endoscopy. However, extensive extraintestinal air dissemination, particularly pneumopericardium, is rare and may raise concern for potentially life-threatening cardiopulmonary complications. Optimal management remains controversial, especially in clinically stable patients.

**Case presentation:**

We report a 37-year-old man with no known cardiopulmonary comorbidities who developed abdominal distension and chest tightness shortly after endoscopic resection of a descending colon lesion at a regional referral centre. The lesion measured approximately 1.0 × 1.2 cm and was treated with snare-based endoscopic resection after submucosal injection, followed by clip closure. On admission, examination revealed abdominal distension and subcutaneous crepitus. Laboratory testing showed leukocytosis, while computed tomography demonstrated pneumoperitoneum, pneumomediastinum, pneumopericardium, and subcutaneous emphysema. The patient remained hemodynamically stable without features of cardiac tamponade. Colonoscopic perforation with extensive air dissemination was diagnosed. In the absence of peritonitis or circulatory compromise, conservative management was initiated, including bowel rest, gastrointestinal decompression, high-flow oxygen, and antibiotics. The patient improved, with follow-up imaging showing marked resolution, and was discharged without complications.

**Conclusion:**

This case suggests that even extensive extraintestinal air, including pneumopericardium, can be managed conservatively in carefully selected patients who remain hemodynamically stable and without peritonitis. Clinical condition should be considered more important than imaging findings when making management decisions.

## Introduction

Colonoscopy is widely used for both diagnostic and therapeutic purposes and is generally considered a safe procedure (1–3). With the increasing use of therapeutic colonoscopic techniques for colorectal lesions, the scope of minimally invasive treatment has expanded considerably (4–7). However, these procedures may still be associated with adverse events, with perforation remaining one of the most serious and potentially dangerous complications (6–9). Although the overall incidence of colonoscopic perforation is low (1–3, 10), it may lead to significant morbidity if not promptly recognized and appropriately managed (8, 9). Early identification and appropriate risk stratification are therefore essential to optimize outcomes and minimize complications in these patients (8, 11).

Beyond pneumoperitoneum, colonic perforation can occasionally result in the dissemination of air into multiple anatomical compartments, including the retroperitoneum, mediastinum, subcutaneous tissue, and, in rare instances, the pericardial space (12–14). This phenomenon is generally attributed to the continuity of fascial planes, which allow air to track from the abdomen into the thoracic cavity (12, 13). Pneumopericardium following colonoscopy is exceedingly rare, and only a limited number of cases have been reported to date (14, 15). Despite its rarity, its presence may raise concern for life-threatening complications such as cardiac tamponade (12), posing a clinical challenge in determining the most appropriate management strategy.

In clinical practice, distinguishing patients who require urgent intervention from those who can be managed conservatively can be difficult, particularly when imaging findings appear alarming (9, 11). The optimal management of colonoscopic perforation complicated by extensive extraintestinal air, particularly when the pericardial space is involved, remains a matter of debate (8, 9). While surgical intervention is often considered in cases of suspected perforation, conservative treatment may be feasible in carefully selected patients without signs of peritonitis or hemodynamic instability (6, 9).

Here, we report a rare case of colonoscopic perforation following snare-based colorectal endoscopic resection that was complicated by extensive air dissemination involving the peritoneal, mediastinal, and pericardial spaces. Notably, the patient was successfully managed with conservative treatment despite the presence of pneumopericardium. This case provides insight into the potential pathways of air spread and further supports the role of nonoperative management in selected patients under close clinical observation. It also highlights the importance of integrating clinical assessment with imaging findings when formulating management strategies.

### Case presentation

This case report is presented in accordance with the CARE guidelines. A 37-year-old man with no known cardiopulmonary comorbidities was admitted to our hospital on March 17, 2026, with abdominal distension and chest tightness that developed several hours after undergoing endoscopic evaluation for hematochezia at a regional referral centre. The patient had no significant past medical history, including hypertension, diabetes mellitus, coronary artery disease, or chronic respiratory conditions. There was no history of prior surgeries, and he was not on any regular medications, including anticoagulants or antiplatelet therapy. He denied any known drug allergies, had no relevant family history of cardiovascular or other chronic diseases, and reported no history of smoking or alcohol consumption. His functional status was independent, with no limitations in daily activities.

Prior esophagogastroduodenoscopy revealed chronic atrophic gastritis. Colonoscopy at the referring centre identified multiple lesions involving the transverse colon, descending colon, sigmoid colon, and rectum. According to the original endoscopy report, the lesion in the descending colon measured approximately 1.0 × 1.2 cm and was suspected to represent a neoplastic lesion. The lesion was treated by snare-based endoscopic resection after submucosal injection with methylene blue mixed saline, which achieved satisfactory lifting. A disposable snare was first used to circumferentially resect the lesion, followed by snare electrocautery resection. The post-resection defect was closed with metal clips, and no active bleeding was noted at the end of the procedure. No intraprocedural perforation was reported in the available records. Additional colonic lesions were also treated endoscopically during the same session. Because the procedure was performed at the referring centre, several procedural details were not available. Specifically, the type of insufflation gas, sedation or anaesthesia method, and total procedure duration were not documented. In addition, the exact number and type of clips used, as well as whether alternative closure methods such as over-the-scope clips (OTSC) or endoloop were considered, could not be verified retrospectively. Shortly after the procedure, the patient developed progressive abdominal distension accompanied by chest tightness. He denied nausea, vomiting, or severe abdominal pain and subsequently presented to our emergency department.

**Figure 1:**
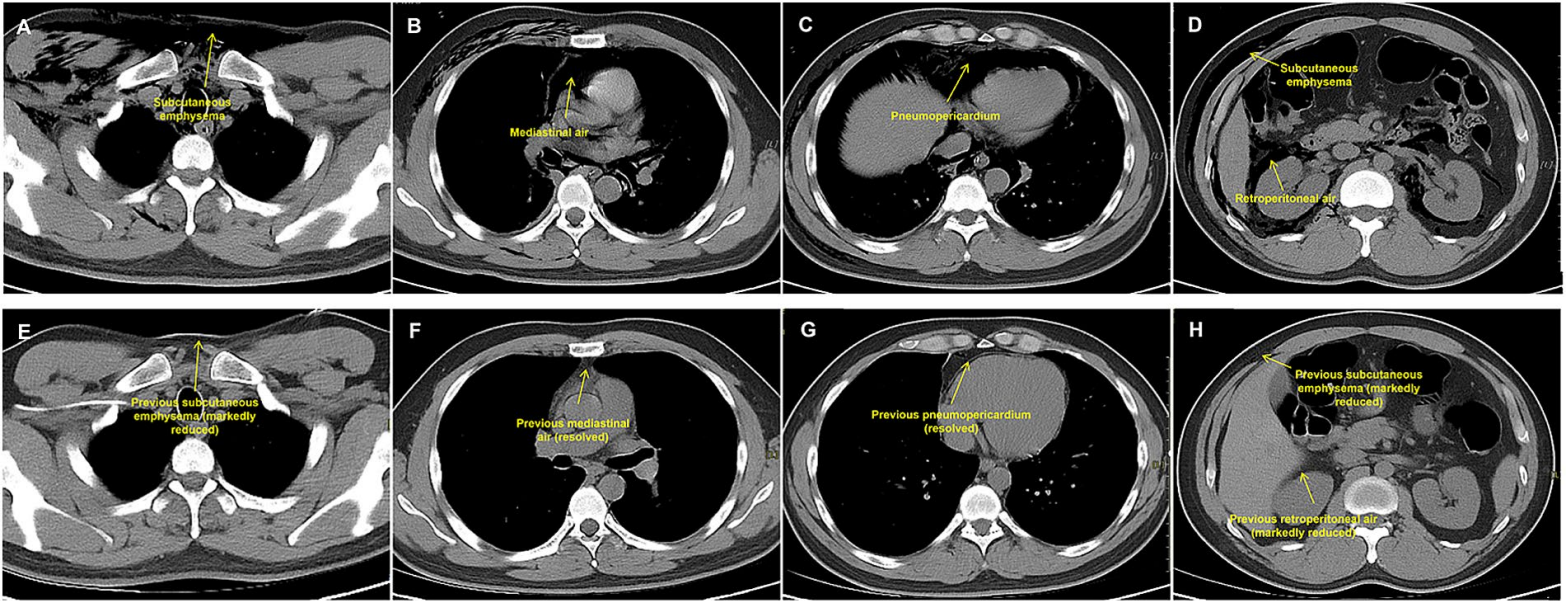
Serial axial CT images demonstrating the distribution and resolution of extraintestinal air following colonic perforation. The upper row **(A–D)** shows images obtained on March 17, 2026, demonstrating air in multiple compartments, including pneumomediastinum, pneumopericardium, and retroperitoneal air. The lower row **(E–H)** shows corresponding anatomical levels on March 24, 2026, with marked interval resolution of these air collections. Arrows indicate the locations of extraintestinal air in each compartment. CT images were reviewed using mediastinal window settings (window width 450 HU, window level 45 HU). Written informed consent for publication of the images was obtained from the patient.

On admission, his temperature was 37.3 °C, heart rate 106 beats/min, respiratory rate 21 breaths/min, and blood pressure 139/72 mmHg. Physical examination revealed abdominal distension with mild epigastric tenderness but no rebound tenderness or guarding. Subcutaneous crepitus was palpable over the neck and right chest wall. Breath sounds were coarse bilaterally. Laboratory tests demonstrated leukocytosis (14.94 × 10^9^/L) with marked neutrophilia (94.8%). Serum lactate (0.8 mmol/L), procalcitonin (<0.1 ng/mL), and cardiac troponin I were within normal limits. Arterial blood gas analysis showed normal acid–base status with adequate oxygenation. Other biochemical and coagulation parameters were largely unremarkable. Emergency computed tomography (CT) of the chest and abdomen revealed extensive pneumoperitoneum, pneumomediastinum, pneumopericardium, and widespread subcutaneous emphysema (Figure 1). Electrocardiography showed sinus rhythm with nonspecific T-wave changes in leads III and aVF and incomplete right bundle branch block. These findings were considered nonspecific and, in the absence of prior ECG for comparison, did not suggest acute right ventricular strain or clinically significant cardiac compromise. Transthoracic echocardiography was technically limited due to intrathoracic air and was therefore non-diagnostic for the assessment of cardiac tamponade. Clinically, however, the patient remained hemodynamically stable without signs of circulatory compromise. No clinical findings suggestive of clinically significant cardiac tamponade were observed.

Given the presence of pneumopericardium and the non-diagnostic echocardiographic assessment, close serial monitoring was maintained throughout the admission to detect any early signs of evolving tension pneumopericardium or cardiac tamponade. Serial chest radiographs were obtained at approximately 24–48 h intervals; these did not demonstrate progressive enlargement of the cardiac silhouette or any radiographic features suggestive of increasing pericardial air volume or cardiac compression. Vital signs were monitored continuously. Blood pressure remained stable throughout the admission at approximately 110–131/71–84 mmHg, and heart rate showed a gradual decline from 106 beats/min on admission to 71 beats/min by the time of discharge, without evidence of progressive tachycardia. No clinical features of Beck’s triad (hypotension, muffled heart sounds, or elevated jugular venous pressure) were identified at any point during the admission. Pulsus paradoxus was not formally measured; however, blood pressure remained consistently stable without any suggestion of paradoxical pulse on bedside assessment. Taken together, the serial clinical, haemodynamic, and radiographic findings supported the absence of evolving tamponade physiology throughout the patient’s hospital course.

Based on the recent history of therapeutic colonoscopy and the imaging findings, a diagnosis of colonoscopic perforation with extensive extraintestinal air dissemination was established. The perforation was considered most likely related to the descending colon resection site; however, the exact source could not be definitively confirmed. A surgical consultation was obtained. In view of the patient’s stable hemodynamic status, the absence of diffuse peritonitis, and the lack of radiological evidence of fluid collection or ongoing contamination, conservative management was pursued. The patient was kept nil per os and received supplemental humidified oxygen therapy [fraction of inspired oxygen (FiO_2_) 0.3; flow rate, 30 L/min] for approximately 72 h. This was administered with the theoretical aim of increasing the inspired oxygen fraction to potentially facilitate nitrogen resorption from the extraluminal air spaces, as has been described in the management of pneumothorax and pneumoperitoneum. However, we acknowledge that direct evidence supporting this approach in the specific setting of post-colonoscopy pneumopericardium is limited, and the oxygen concentration used in this case was lower than that employed in dedicated nitrogen washout protocols. Gastrointestinal decompression was maintained for 3 days. Broad-spectrum intravenous antibiotics (piperacillin–tazobactam, 4.5 g every 8 h) were administered for 7 days, given the concern for possible microperforation and contamination risk, although shorter durations may be sufficient in selected clinically stable cases. Supportive care included intravenous fluids, electrolyte management, and parenteral nutrition via a peripherally inserted central catheter. This approach was chosen given the anticipated duration of bowel rest and to maintain adequate nutritional support during the acute phase. Venous thromboembolism prophylaxis was provided with low-molecular-weight heparin together with mechanical measures. The patient was closely monitored with serial clinical assessments and laboratory testing.

**Figure 2:**
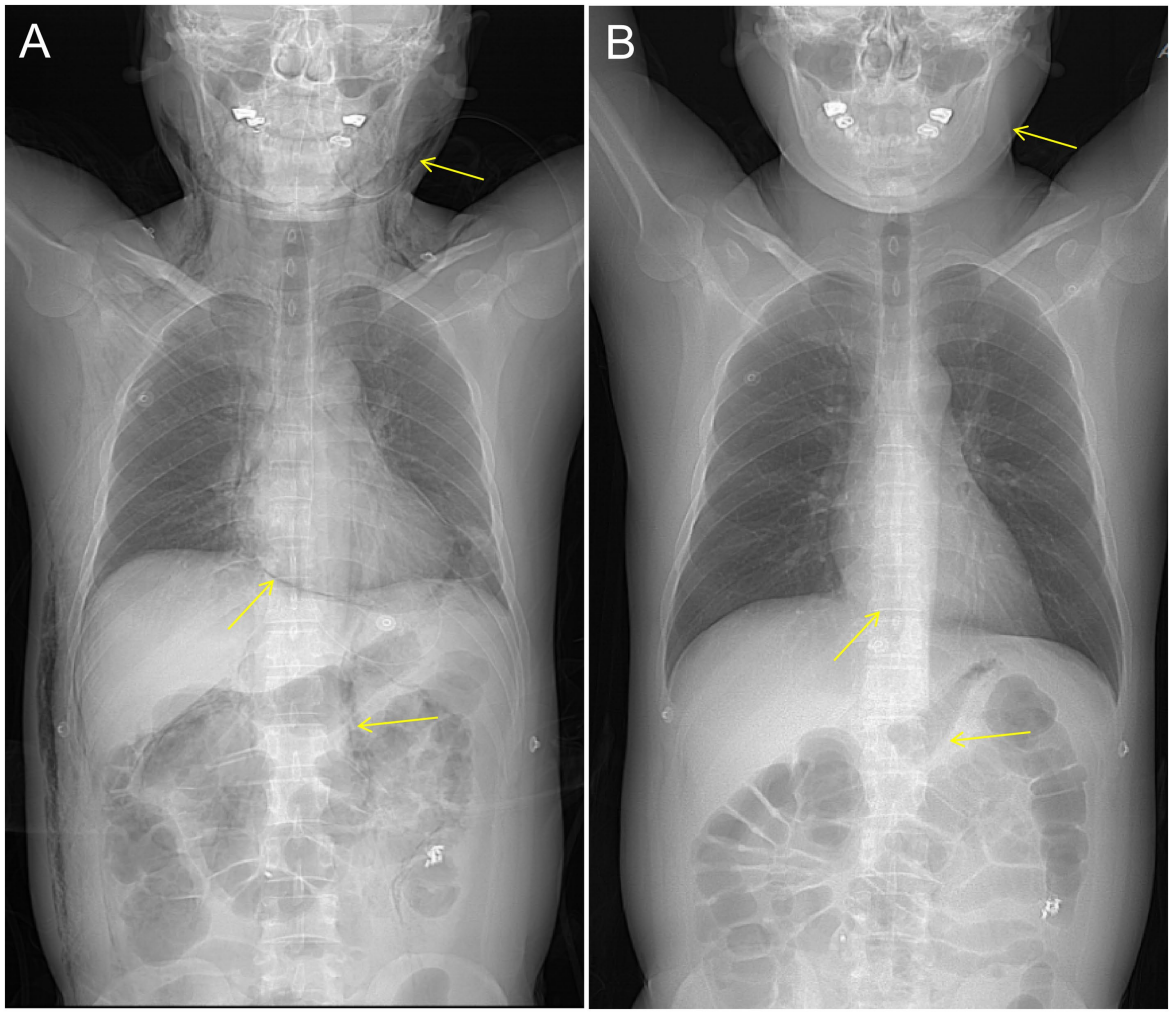
Serial chest and abdominal radiographs illustrating the resolution of extraintestinal air following colonic perforation. **(A)** Initial radiograph (March 17, 2026) showing increased radiolucency in the abdominal cavity, indicative of pneumoperitoneum. Arrows indicate the regions of free air. **(B)** Follow-up radiograph (March 24, 2026) showing significant resolution of intraperitoneal free air. Arrows indicate the corresponding regions on follow-up imaging, demonstrating marked interval resolution. Written informed consent for publication of the images was obtained from the patient.

**Figure 3:**
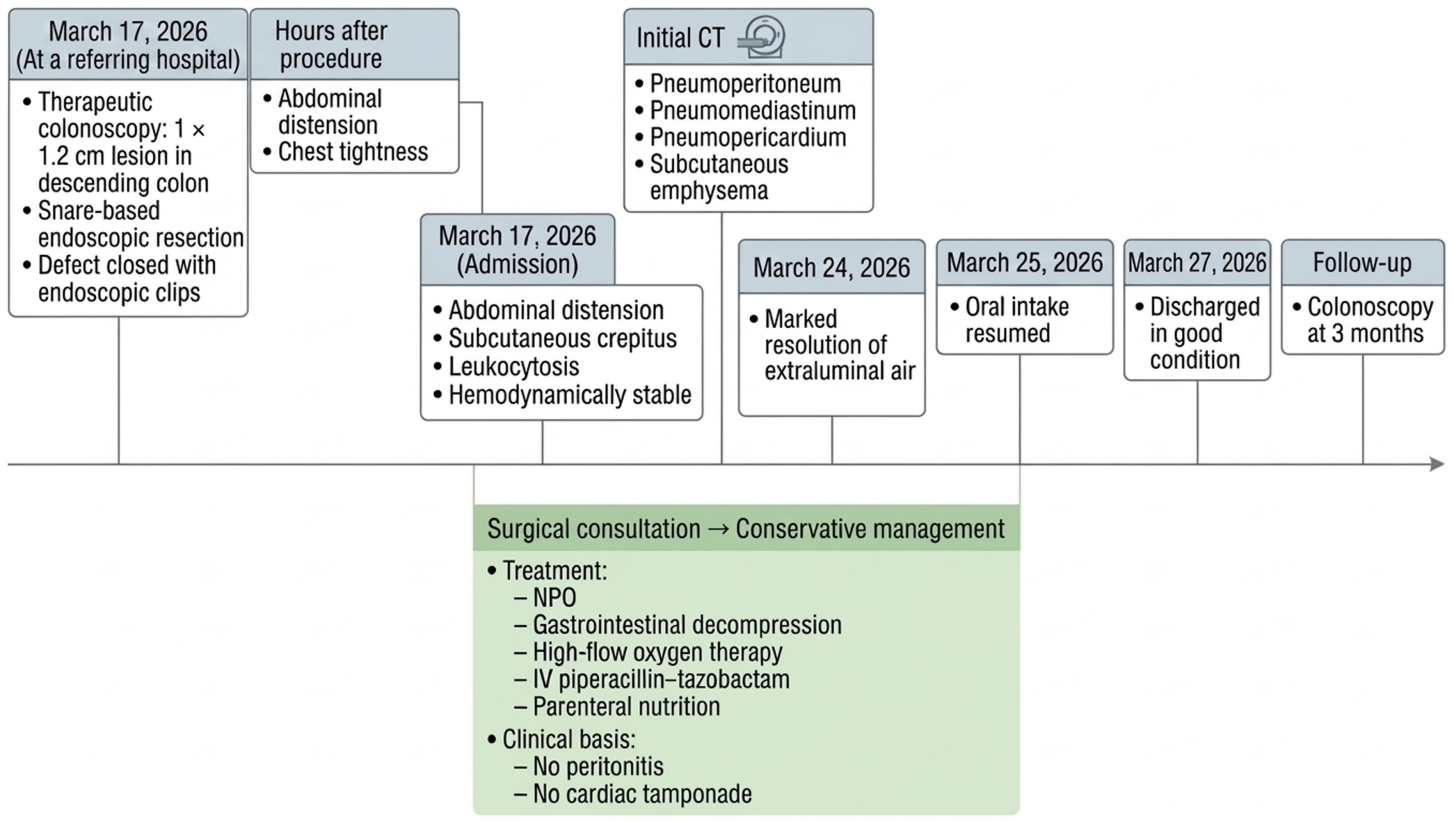
Timeline of the patient’s clinical course. After therapeutic colonoscopy with snare-based endoscopic resection at a referring hospital, the patient developed abdominal distension and chest tightness. Imaging revealed extensive extraluminal air involving the peritoneal, mediastinal, pericardial, and subcutaneous compartments. Given stable clinical status without peritonitis or clinical evidence of hemodynamically significant cardiac tamponade, conservative management was pursued after surgical consultation. The patient improved steadily, with marked radiological resolution by March 24, 2026, resumption of oral intake on March 25, 2026, and discharge on March 27, 2026. Follow-up colonoscopy was scheduled for 3 months post-discharge.

During hospitalization, the patient remained clinically stable, with no evidence of peritonitis or systemic infection. His symptoms gradually improved. Oral intake was resumed stepwise beginning on March 25 and was well tolerated. A follow-up CT scan on hospital day 7 demonstrated marked resolution of pneumoperitoneum, pneumomediastinum, pneumopericardium, and subcutaneous emphysema (Figure 1). Serial chest and abdominal radiographs also demonstrated progressive resolution of intraperitoneal free air (Figure 2). Histopathological examination of the descending colon lesion revealed high-grade dysplasia with focal intramucosal carcinoma involving lymphoepithelial structures, with a negative deep resection margin of approximately 2 mm. The patient was discharged on hospital day 11 in good condition. The clinical course is summarized in Figure 3. At discharge, his vital signs were stable, subcutaneous emphysema had resolved, and abdominal examination was unremarkable. He was advised to avoid strenuous activity and to undergo follow-up colonoscopy after 3 months.

## Discussion

Colonoscopy-associated perforation is an uncommon but well-recognized complication, with reported incidence varying according to the complexity of the procedure, particularly in advanced therapeutic colonoscopic interventions (4–6, 10, 16). While pneumoperitoneum is the most common manifestation, the presence of extensive extraintestinal air involving multiple anatomical compartments is considerably less frequent (12, 14). Pneumopericardium following colonoscopic procedures is especially rare, with only a limited number of cases described in the literature (14, 15, 17, 18). As a result, its clinical presentation and optimal management are not yet fully characterized, and most available evidence is derived from isolated case reports (14, 15). To facilitate comparison with the present case, representative previously reported cases of post-colonoscopy pneumopericardium are summarized in Table 1 (14, 17, 19, 20).

**Table 1:** Representative previously reported cases of post-colonoscopy pneumopericardium and comparison with the present case.

Study	Year	Age/sex	Indication	Suspected site of perforation	Closure method	Insufflation gas	Management	Outcome
Bakker et al.	1991	NR	Colonoscopic polypectomy	NR	Primary closure	NR	Surgical	Recovered
Fitzgerald et al.	1992	NR	Colonoscopy (NR)	NR	NR	NR	Surgical	Recovered
Park et al.	2007	46/F	Colonoscopy (NR)	Sigmoid colon	Endoscopic clips	NR	Endoscopic closure and conservative treatment	Recovered
Nwashilli et al.	2016	68/F	Therapeutic colonoscopy (snare polypectomy)	Rectosigmoid junction	NR	NR	Conservative treatment	Recovered
Wu et al.	2026	37/M	Therapeutic colonoscopy (snare-based resection)	Descending colon	Endoscopic clips	NR	Conservative treatment	Recovered

Representative cases of pneumopericardium following colonoscopy or colonoscopic therapeutic intervention identified from the literature are summarized and compared with the present case. Reported variables include demographic characteristics, procedural indication, suspected site of perforation, closure method, insufflation gas, management, and outcome. Only reports explicitly documenting post-colonoscopy pneumopericardium were included. NR, not reported.

**Figure 4:**
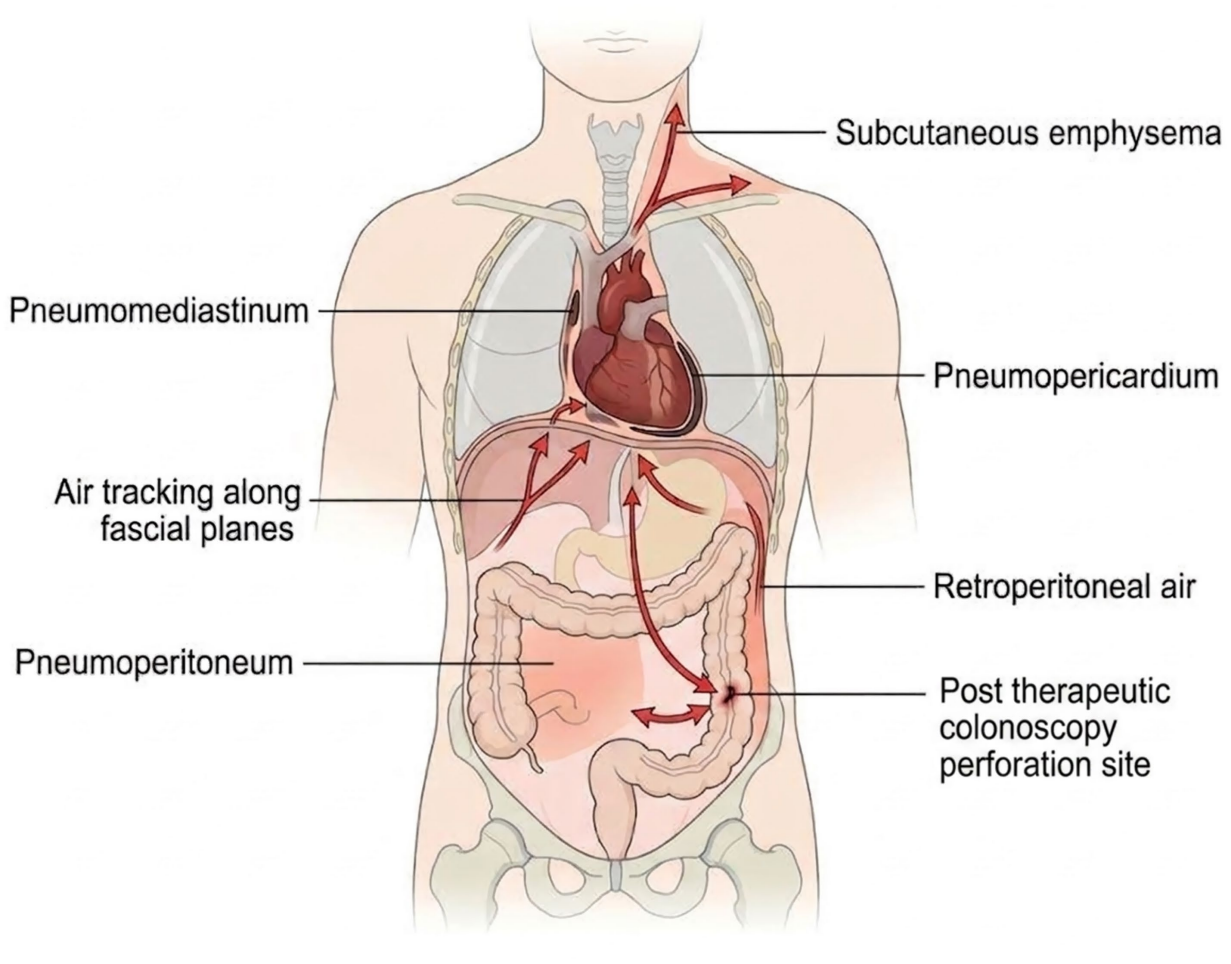
Schematic diagram illustrating the potential pathways of air dissemination in this case. Following colonic perforation after endoscopic resection, insufflated air may enter the peritoneal and retroperitoneal spaces. Air within the retroperitoneum can ascend along fascial planes into the mediastinum, leading to pneumomediastinum, and may further extend into the pericardial sac, resulting in pneumopericardium. Air may also track into the subcutaneous tissues of the neck and chest, producing subcutaneous emphysema.

The spread of air from the colon to the mediastinum and pericardial space is generally attributed to the continuity of fascial planes (12, 13, 21). When the integrity of the colonic wall is disrupted, insufflated air may enter the retroperitoneal space, particularly when the affected segment is partially retroperitoneal, such as the descending colon (12). From there, air can track along vascular sheaths and connective tissue planes into the mediastinum, and in some cases extend further into the subcutaneous tissues and pericardial sac (12, 13, 21–23), resulting in pneumomediastinum, pneumopericardium, and subcutaneous emphysema (Figure 4). This pathway provides a plausible explanation for the simultaneous presence of pneumoperitoneum, pneumomediastinum, subcutaneous emphysema, and pneumopericardium observed in this patient (14). Nonetheless, given the coexistence of intraperitoneal and extraperitoneal air, alternative routes of air migration cannot be entirely excluded. It is also possible that a combination of mechanisms contributed to the observed distribution of air in this case. While this anatomical continuity explains the pattern of air dissemination, the exact source of the leak remains an important consideration. Notably, in the present case, the lesion was relatively small and treated with snare-based endoscopic resection rather than formal submucosal dissection, suggesting that extensive extraintestinal air dissemination may occur even after less extensive colorectal therapeutic endoscopy.

Although the descending colon resection site was considered the most likely source of perforation based on procedural context and anatomical considerations, the exact origin of the extraluminal air cannot be definitively confirmed. Alternative sources should therefore be considered. Microperforation at other lesions treated during the same session (including the transverse colon, sigmoid colon, and rectum) cannot be completely excluded, particularly in the absence of detailed procedural documentation from the referring centre. In addition, barotrauma-related mucosal injury from over-insufflation, particularly when room air is used instead of carbon dioxide (CO₂), which is more rapidly absorbed, and, less likely, rupture of pre-existing pulmonary blebs represent potential alternative mechanisms for the observed air distribution (24, 25). Additional imaging modalities, such as delayed contrast-enhanced CT or water-soluble contrast studies, may be helpful in localizing the site of leakage in similar cases and should be considered when diagnostic uncertainty persists. However, further imaging was not pursued in this case due to the patient’s stable clinical condition and the decision to proceed with conservative management.

The clinical relevance of pneumopericardium lies in its potential to progress to cardiac tamponade (12). However, the presence of air within the pericardial space does not invariably translate into hemodynamic compromise (12). In this case, despite extensive air dissemination, the patient remained hemodynamically stable, and no clinical findings suggestive of clinically significant cardiac tamponade were observed. Echocardiographic assessment was technically limited by intrathoracic air and therefore could not reliably exclude tamponade on imaging grounds alone. This observation underscores the importance of correlating imaging findings with the overall clinical condition rather than relying on radiological severity alone (8, 11). Close monitoring remains essential, as clinical status may evolve even when initial findings appear reassuring.

The management of colonoscopic perforation accompanied by extensive extraintestinal air remains challenging and is often individualized (8, 9). Surgical intervention is generally indicated in the presence of generalized peritonitis, ongoing leakage, or clinical deterioration. In contrast, a more conservative approach may be appropriate in selected patients who remain stable and show no signs of diffuse peritoneal irritation or systemic infection (8, 9). In the present case, the decision to pursue conservative management was supported by the absence of hemodynamic instability, generalized peritonitis, or radiologic evidence of ongoing contamination, such as intra-abdominal fluid collection. These findings are consistent with current guideline recommendations for the nonoperative management of selected colonoscopic perforations. Under close monitoring, this approach resulted in gradual clinical and radiological resolution without the need for surgical intervention, and the decision was primarily supported by the absence of clinical signs of hemodynamic compromise rather than definitive echocardiographic exclusion of tamponade.

High-flow supplemental oxygen was administered in this case with the theoretical rationale of promoting nitrogen resorption from extraluminal air collections by elevating the inspired oxygen concentration. This strategy has been described in the management of spontaneous and iatrogenic pneumothorax and extrapolated to post-procedural pneumoperitoneum; however, evidence specifically supporting its use in pneumopericardium is limited to anecdotal case-level observations. Furthermore, the FiO₂ achieved in the present case (0.3) is lower than the high-concentration oxygen typically used in nitrogen washout protocols, and its specific contribution to the resolution of pericardial air in this patient cannot be determined. Whether the observed radiological resolution reflects the effect of supplemental oxygen, spontaneous resorption, or a combination of both remains uncertain. We therefore consider this intervention to be of uncertain but potentially adjunctive benefit, and encourage future case reports to document oxygen parameters and timing of resolution to better characterize the role of supplemental oxygen in this setting.

This case highlights several clinically relevant considerations. Extensive extraintestinal air, even when involving the pericardial space, does not necessarily mandate immediate invasive intervention in the absence of clinical deterioration. This may also apply in patients undergoing relatively limited endoscopic resection, provided that close monitoring excludes peritonitis, ongoing leakage, or hemodynamic compromise. Careful and dynamic assessment remains essential, and management decisions should be guided by the overall clinical picture rather than imaging findings alone (8, 11). In this context, multidisciplinary evaluation may further support appropriate decision-making and help avoid unnecessary procedures.

Building on these considerations, conservative management may be considered in carefully selected patients. In the present case, this approach was supported by a combination of factors: stable hemodynamic status, absence of peritonitis, reassuring laboratory parameters (normal lactate, low inflammatory markers), absence of radiologic evidence of ongoing contamination (no significant fluid collection on CT), successful endoscopic closure of the defect (26), and the availability of close monitoring in an intensive care setting with immediate surgical backup. These findings underscore the importance of individualized clinical assessment when determining management strategies (8, 9, 12–14). A limitation of this report is that the index procedure was performed at a referring centre, and several procedural details—including insufflation method and procedural duration—were not available for retrospective review.

### Patient perspective

The patient reported that the sudden onset of abdominal distension and chest tightness caused significant anxiety, particularly after being informed of the extensive findings on CT imaging. During hospitalization, he was concerned about the possibility of requiring surgical intervention and potential long-term complications. As his symptoms improved with conservative management, his confidence in the treatment approach increased. At follow-up, he expressed satisfaction with the recovery process and relief that invasive procedures were avoided. He also reported good tolerance of resumed oral intake and no recurrent symptoms.

## Data Availability

The original contributions presented in the study are included in the article/Supplementary material, further inquiries can be directed to the corresponding authors.
